# Emotional and behavioral problems in late preterm and early term births: outcomes at child age 36 months

**DOI:** 10.1186/s12887-016-0746-z

**Published:** 2016-12-01

**Authors:** Kim Stene-Larsen, Astri M. Lang, Markus A. Landolt, Beatrice Latal, Margarete E. Vollrath

**Affiliations:** 1 Department of Mental Health and Suicide, Domain of Mental and Physical Health, Norwegian Institute of Public Health, Postbox 4404, Nydalen, 0403 Oslo, Norway; 2 Department of Neonatal Intensive Care, Women and Children’s Division, Oslo University Hospital, Oslo, Norway; 3 Department of Psychosomatics and Psychiatry, University Children’s Hospital Zurich, Zurich, Switzerland; 4 Child Development Center, University Children’s Hospital Zurich, Zurich, Switzerland; 5 Psychological Institute, University of Oslo, Oslo, Norway

**Keywords:** Late preterm, Early term, Gender, Emotional problems, Behavioral problems, Internalizing problems, Externalizing problems

## Abstract

**Background:**

Recent findings has shown that late preterm births (gestational weeks 34–36) and early term births (gestational weeks 37–38) is associated with an increased risk of several psychological and developmental morbidities. In this article we investigate whether late preterm and early term births is associated with an increased risk of emotional and behavioral problems at 36 months of age and whether there are gender differences in risk of these outcomes.

**Methods:**

Forty-three thousand, two hundred ninety-seven children and their mothers participating in the Norwegian Mother and Child Cohort Study (MoBa). One thousand, eight hundred fifty-three (4.3%) of the children in the sample were born late preterm and 7,835 (18.1%) were born early term. Information on gestational age and on prenatal and postnatal risk factors was retrieved from the Medical Birth Registry of Norway. Information on emotional and behavioral problems was assessed by standardized questionnaires (CBCL/ITSEA) filled out by the mothers. Gender-stratified logistic regression analyses were used to explore the association between late preterm / early term and emotional and behavioral problems at 36 months of age.

**Results:**

We found a gender-specific increased risk of emotional problems in girls born late preterm (OR 1.47 95%CI 1.11–1.95) and in girls born early term (OR 1.21 95%CI 1.04–1.42). We did not find an increased risk of emotional problems in boys born late preterm (OR 1.09 95%CI 0.82–1.45) or early term (OR 0.93 95%CI 0.79–1.10). Behavioral problems were not increased in children born late preterm or early term.

**Conclusion:**

Girls born late preterm and early term show an increased risk of emotional problems at 36 months of age. This finding suggests that gender should be taken into account when evaluating children born at these gestational ages.

## Background

Premature birth is associated with a range of developmental and psychological morbidities [1–4]. It has also been established that the more premature the child, the more frequent and severe these morbidities [3–6]. Even though the risk of morbidities is less for children born late preterm (gestational weeks 34-36) and early term (gestational weeks 37–38) compared with children with shorter gestational ages [7] recent findings show increased rates of a range of outcomes such as communication impairments, cerebral palsy, ADHD, intellectual disabilities, and emotional and behavioral problems also in these children [2, 8–13]. In addition, gender-specific outcomes in impairments have been suggested. For example, girls born moderately preterm (gestational weeks 32–35) are reported to have an increased risk of emotional and behavioral problems. [9] The mechanisms behind this gender-specific risk are not well understood and there is no data concerning whether a similar pattern exists for children born late preterm or early term.

With the rapidly increasing population of early term and late preterm births [14–16], it is of clinical and public health importance to map the potential gender-specific psychological outcomes in this large population. As potential effects are most likely small, studies addressing this question will need large population-based cohorts. Investigating emotional and behavioral problems / morbidities early in a child’s life sequelae is especially important given that early onset problems often persist into later childhood and adolescence, with lasting impact on later development and social competencies [17–19]. An important issue in research on psychological sequalae in children born preterm is whether shorter gestation length is a risk factor per se or whether health risks associated with preterm birth—confounders—are responsible for later adverse outcomes. For example, many children born preterm are small for their gestational age, which in turn is a risk factor for later neurobehavioral problems. [20] Therefore, it is necessary to carefully control for all known risk factors associated with both preterm birth and psychological problems in the child.

We thus present a case cohort study including more than 43 000 children, in which we addressed whether late preterm and early term births are associated with an increased risk of emotional and behavioral problems at age 36 months. We also determined potential gender differences in the risk of emotional and behavioral problems among these children.

## Method

### Study population

This study is based on data from the Norwegian Mother and Child Cohort study (MoBa), a prospective population-based pregnancy cohort study conducted by the Norwegian Institute of Public Health (http://www.fhi.no/morogbarn) [21]. Participants were recruited from all over Norway from 1999 to 2008, and 38.7% of the women invited agreed to participate. The cohort now includes 109 000 children, 91 000 mothers and 71 700 fathers [22]. The women completed questionnaires at the 17th and 30th weeks of pregnancy and at child ages 6, 18, and 36 months. The response rates among mothers who consented to join the study were 95 and 92% at gestational weeks 17 and 30, and 87, 77, and 62% at child ages 6, 18 and 36 months, respectively [21, 22]. In addition, information on maternal age, length of the pregnancy, and pre- and postnatal risk factors was retrieved from the Medical Birth Registry of Norway (MBRN) [23].

The MoBa study and access to data from the MBRN was approved by the Regional Committee for Medical Research Ethics in Norway (S-97045 & S-95113) and by the Norwegian Data Protection Authority (01/4325).

### Inclusion and exclusion criteria

The inclusion criteria for this study were that the mother had completed the questionnaires at gestational week 17 (*N* = 101 624), and at child age 36 months (*N* = 49 504). Of the 49 504 participants who fulfilled the inclusion criteria, we excluded children with severe malformations or syndromes (*N* = 1488), severe hearing deficits (*N* = 163), and cerebral palsy (*N* = 63). We also excluded children with gestations longer than 41 weeks 6 days, or shorter than 33 weeks 6 days (*N* = 4316). The final sample comprised 43 297 children, of whom 1853 (4.3%) were born late preterm and 7835 (18.1%) were born early term. The proportions of infants born early term and late preterm in our study correspond roughly to those found in the Medical Birth Registry of the total Norwegian population with 5.2% infants born between gestational weeks 28 and 36 and 17.4% born between gestational weeks 37 and 38.

### Measures

#### Predictors

Information on gestational age based on ultrasound examination was retrieved from the MBRN. In accordance with established international definitions [24], we chose for the purpose of the current study to discriminate between early term birth (gestational age 37 weeks 0 days – 38 weeks 6 days) and term birth with a gestational age of 39 weeks 0 days to 41 weeks 6 days. Late preterm birth was defined as a gestational length of 34 weeks 0 days to 36 weeks 6 days.

#### Emotional and behavioral problems

Emotional and behavioral problems at age 36 months were assessed through maternal ratings of 17 items from the Child Behavior Check List (CBCL) and 12 items from the Infant Toddler Social and Emotional Assessment (ITSEA) [25, 26]. The CBCL and the ITSEA strongly overlap with respect to the underlying concepts of internalizing (emotional) and externalizing (behavioral) problems that they both measure. In line with previous research [27], we combined the internalizing items and the externalizing items from the CBCL and the ITSEA, respectively, into two new scales. Tables 1 and 2 show the items and the questionnaires they originate from. The reliability was found to be high for both scales with ordinal thetas of 0.81 for the internalizing scale and 0.90 for the externalizing scale [27]. Both scales were highly skewed, and an item response analytic approach [28] was used to define the cut-off point with the highest discriminate ability for each scale. This cut-off point was found to be close to the 95th percentile for both the externalizing and the internalizing scale in the total sample and among girls. For boys, we found no cut-off point that was better than any other so we tested several (85, 90, 95 and 98%) leading to roughly the same overall results. For simplicity (and because the results were roughly the same using differing cut-off points), we choose to set the cut-off as close as possible to the 95th percentile in all analyses.

**Table 1:** Items included in the emotional problems scale

Item	Original scale
Gets too upset when separated from parents	CBCL
Clings to adults or too dependent	CBCL
Disturbed by any change in routine	CBCL
Sudden changes in mood or feelings	CBCL
Too fearful or anxious	CBCL
Is very anxious about getting dirty	ITSEA
Wants things to be clean and tidy	ITSEA
Places toys or other objects in a certain order/sequence over and over again	ITSEA
Wakes up in the night and needs help to get back to sleep	ITSEA
Gets distressed when you go out and he/she is going to be looked after by family or a babysitter he/she knows	ITSEA
Seems to have less fun than other children	ITSEA
Seems to be unhappy, sad, or depressed	ITSEA

**Table 2:** Items included in the behavioral problems scale

Item	Original scale
Canʼt concentrate, canʼt pay attention for long	CBCL
Canʼt sit still, restless, or hyperactive	CBCL
Canʼt stand waiting, wants everything now	CBCL
Defiant	CBCL
Demands must be met immediately	CBCL
Doesnʼt seem to feel guilty after misbehaving	CBCL
Gets in many fights	CBCL
Gets into everything	CBCL
Hits others	CBCL
Poorly coordinated or clumsy	CBCL
Punishment doesnʼt change his/her behavior	CBCL
Quickly shifts from one activity to another	CBCL
“Tests” other children to see whether they get angry	ITSEA
Becomes aggressive when he/she is frustrated	ITSEA
Hits, shoves, kicks, and bites other children (not including siblings)	ITSEA
Is disobedient or defiant (e.g., refuses to do anything you ask)	ITSEA
Is extremely noisy. Shouts and screams a lot	ITSEA

#### Control variables

Prenatal risk: An index of prenatal risk was computed by counting the number of the following risk factors present: Maternal gestational diabetes, preeclampsia/HELLP syndrome (severe preeclampsia), multiple gestations, and being small for gestational age (SGA). SGA was coded by combining the infant’s birth weight and gestational age according to established norms. [29] Information on prenatal risk factors was retrieved from the MBRN.

Cesarean section: Information on cesarean section (elective or emergency) was retrieved from the MBRN. In the analyses, only emergency cesarean section was included as a risk factor, because this mode of delivery is the factor that is associated the most with an increased risk of complications [30].

Postnatal risk: An index of postnatal risk factors for the child was computed by counting the presence of the following risk factors present in the child: a 5 min Apgar [31] score of 6 or less; a diagnosis of respiratory distress or intracranial bleeding; and mechanical ventilation after birth. Information on all postnatal risk factors was retrieved from the MBRN.

### Statistical analysis

SPSS Statistics Version 20 (SPSS Inc., Chicago IL) was used for the statistical analyses whereas R-statistics was used for the IRT analyses of the emotional and behavioral scales. Logistic regression analyses were used to explore the unadjusted and adjusted associations between early term/late preterm birth and emotional and behavioral problems at child age 36 months. Gender differences in risk of emotional and behavioral problems in children born early term and late preterm were explored in stratified samples. In addition we performed in depth analyses of the gender differences with the interaction terms (gender (0 = boys) * early term) and (gender (0 = boys) * late preterm) included in the regression analyses of the total sample. The percentage of missing values in the majority of variables was low (0.4 to 2%). In contrast, the percentage of missing values exceeded 5% on the maternal education variable. To substitute the number of missing values in this variable, we performed a maximum likelihood imputation procedure using information from the highly correlated variables of maternal and partner income [32].

#### Sensitivity analyses

In the analyses presented in this article we restricted the confounders to the prenatal and postnatal variables that we had information on. However, in order to fully explore the impact of other potentially confounding variables we also conducted logistic regression analyses including maternal level of education and income, maternal age, and maternal anxiety and depression (SCL-8) at childs age 36 months.

## Results

The demographic and perinatal characteristics of the sample have been presented previously [11]. In general, children born late preterm and early term differed from term born children on a variety of characteristics. Importantly, mothers were less educated, they were older, and the prevalence of prenatal risk factors such as gestational diabetes and small for gestational age was higher [11].

### Logistic regression analyses

Table 3 shows the unadjusted and adjusted associations between late preterm, and early term, births and emotional and behavioral problems at 36 months in the entire cohort, whereas Table 4 shows the gender-stratified analyses. Children born late preterm had 40% higher odds of having emotional problems than the children born at term in the unadjusted analysis. After controlling for confounders, their odds were still 26% higher than the odds for children born at term. At the same time, neither children born late preterm nor children born early term had elevated odds for behavioral problems.

**Table 3:** Logistic regression analysis showing the associations between early term and late preterm birth, and child emotional and behavioral problems at 36 months of age

	Emotional problems		Behavioral problems	
	Unadjusted OR (95%CI)	Adjusted OR (95%CI)	Unadjusted OR (95%CI)	Adjusted OR (95%CI)
Term birth	Reference	Reference	Reference	Reference
Early term	1.09 (0.98–1.22)	1.07 (0.96–1.98)	1.00 (0.90–1.14)	1.00 (0.90–1.14)
Late preterm	1.40*** (1.17–1.68)	1.26* (1.04–1.52)	1.16 (0.97–1.14)	1.10 (0.91–1.33)

Note: Adjusted analyses include information on gestational diabetes, preeclampsia/HELLP syndrome, multiple gestation, small for gestational age, 5 min APGAR < 6, respiratory distress, intracranial bleeding and mechanically ventilated; * *p* < .05; *** *p* < .001

**Table 4:** Gender-stratified logistic regression analysis showing the unadjusted and adjusted associations between early term/late preterm birth, and behavioral problems at child age 36 months

	Boys				Girls			
	Emotional problems		Behavioral problems		Emotional problems		Behavioral problems	
	Unadjusted OR (95%CI)	Adjusted OR (95%CI)	Unadjusted OR (95%CI)	Adjusted OR (95%CI)	Unadjusted OR (95%CI)	Adjusted OR (95%CI)	Unadjusted OR (95%CI)	Adjusted OR (95%CI)
Term birth	Reference	Reference	Reference	Reference	Reference	Reference	Reference	Reference
Early term	0.99 (0.85–1.15)	0.95 (0.83–1.12)	0.97 (0.84–1.11)	0.96 (0.84–1.11)	1.22** (1.05–1.41)	1.20** (1.03–1.39)	1.06 (0.91–1.24)	1.05 (0.90–1.23)
Late preterm	1.21 (0.93–1.57)	1.06 (0.81–1.39)	1.14 (0.89–1.44)	1.09 (0.85–1.39)	1.64*** (1.27–2.11)	1.52** (1.16–1.99)	1.20 (0.90–1.59)	1.12 (0.84–1.51)

Note: Adjusted analyses include information on gestational diabetes, preeclampsia/HELLP syndrome, multiple gestation, small for gestational age, 5 min APGAR < 6, respiratory distress, intracranial bleeding and mechanically ventilated; * *p* < .05; ** *p* < .01; *** *p* < .001

In the gender-stratified analyses (Table 4), we found that girls born late preterm had 64% higher odds of emotional problems compared with girls born at term. Adjusting for the confounders slightly reduced the odds to 52%. We also found that girls born early term had 22% increased odds of emotional problems and 20% increased odds in the adjusted analysis. We found no increased odds of emotional or behavioral problems among boys born late preterm or early term.

### In depth analyses of the gender specific outcome

The interaction terms (gender (0 = boys) * early term) and (gender (0 = boys) * late preterm) included in the fully adjusted analyses of the total sample were both significant with odds ratios of 1.18 (95%CI 1.01–1.38) and 1.40 (95%CI 1.06–1.86).

### Results from the sensitivity analyses

Including maternal level of education and income, maternal age, and maternal anxiety and depression in the fully adjusted logistic regression analyses had little impact on the overall results presented. For children born late preterm the increased risk of emotional problems at 36 months was reduced from 26 to 25%. In a similar manner the increased risk of emotional problems in girls born early term was reduced from 20 to 18%. The largest observed change was found among girls born late preterm with a reduction in risk from 52 to 47% in the sensitivity analyses.

## Discussion

Our study had two main goals: first, to investigate the association between late preterm/early term births and emotional and behavioral problems at child age 36 months, and second, to explore the role of gender on these outcomes.

In line with previous research in older children [12] we found that children born late preterm had an increased risk of emotional problems at 36 months of age. This finding implies that emotional problems associated with late preterm births may be present already at a very early age. On the other hand, we found no evidence of increased risk of behavioral problems among children born late preterm in our study. This finding contradicts previous studies that have found evidence of both ADHD and other behavioral problems in children born late preterm. [2, 9, 10] A likely explanation for this contradiction could be the young age of the children in our study (36 months) which could have made it difficult to detect behavioral problems that would be more easily recognizable or emerge more frequently at an older age. With regard to children born early term, we found no increased risk of either emotional or behavioral problems at 36 months. Studies that focus on emotional or behavioral problems in children born early term are extremely scarce and, to the best of our knowledge, only one study has investigated this outcome in a group of children aged 2 to 17 years. In that study, an increased risk of behavioral problems was found for children born during gestational week 37 but not for children born during gestational weeks 38 or children born late preterm. [10] A limitation of that study was a limited statistical power with only 171 children born late preterm and 238 born during gestational week 37.

With regards to the gender-stratified analyses, we found that the risk of emotional problems was specific for girls. Moreover, in addition to girls born late preterm, girls born early term also showed an increased risk of emotional problems at 36 months. This finding was further strengthened by the significant interaction terms in the total sample. With regard to gender differences in risk, we have only found two previous studies [9, 33] to compare our findings with. The first study found an increased risk of behavioral and emotional problems in 4-year-old children born moderately preterm and they found that this risk of emotional problems was strongest for girls [9]. In the other study they found that children born moderately preterm had an increased risk of developing behavioral precursors to ADHD at 5 years of age and this risk was strongest for the girls born moderately preterm [33]. Direct comparison of our results with these studies is difficult though as the included gestational weeks of moderately preterm (gestational weeks 32–35) only partly overlap with those included in late preterm (gestational weeks 34–36).

An explanation of the gender differences in risk of emotional problems found in this study could possibly be found in how the genders differ in their early social development. Recent findings have shown that social and structured forms of play emerge systematically earlier in girls than in boys [34]. As an example girls tend to involve in associative play around 3 years of age whereas boys follow a couple of years later. This pattern could be a key to explaining the sex differences observed in our study. First, communication skills are an important element in associative play. In order to effectively interact with same age peers good communication skills is crucial. We have previously documented that children born early term and late preterm have an increased risk of communication impairments. This could imply that these same children are at risk of not succeeding in their social interactions with their peers. Possible psychological reactions could be emotional problems such as sadness, loneliness, overly dependent on parents etc. that was found more frequently amongst the girls born early term and late preterm in our study. Given the gender difference in timing of the development of associative play one would expect girls born early term and late preterm to have an increased risk of emotional problems debuting around 3 years of age whereas one would expect the boys to debut with their symptoms (emotional or behavioral problems) a couple of years later. Unfortunately we do not have the data to test this hypothesis in our data material.

We did not find evidence of behavioral problems at 36 months in children born early term or late preterm. Several studies report that preterm children predominantly display attention problems and not hyperactivity [35]. The behavioral problems measure used in our study primarily focused on symptoms of conduct disorder such as getting into many fights, hitting others, not feeling guilty after misbehaving etc. It is therefore a possibility that we did not manage to detect potential behavioral problems amongst the children born early term and late preterm. On the other hand the measure included some items on attention problems such as not being able to concentrate, not being able to pay attention for long and quickly shifting from one activity to another. These items were tested as a cluster and separately in the preliminary analyses and we did not find any association with early term or late preterm birth.

Our study has some strengths and limitations that should be kept in mind when interpreting our findings. First, to our knowledge it is the largest cohort study ever done on late preterm, early term births and later emotional and behavioral problems. The size of our study sample with more than 1800 children born late preterm and more than 7000 children born early term ensures high statistical power, allowing us to detect even subtle associations with emotional and behavioral outcomes. In addition, detailed information on perinatal risk factors was available through the Medical Birth Registry of Norway, allowing us to adjust for these. With regard to the limitations of the study, the mothers participating in the MoBa study are slightly older (30 vs 29 years), smoke less, and are more compliant with the official health advice such as taking folic acid compared with the average mothers in the Norwegian population [36]. This overrepresentation of “healthy” women may reduce the variance of risk factors and emotional/behavioral problems in the child and thus lead to an underestimation of the true associations. Second, the study did not include a full scale testing of emotional/behavioral problems of the child but was based on maternal ratings on short versions of the CBCL/ITSEA which limits comparison with results from other studies.

### Implications

Early emotional problems in the clinically significant range were found in girls born late preterm and early term. Given the high proportion of births of these two groups, this finding has clinical and public health care relevance. There are three lines of research we suggest, based on our findings. First, we need studies following the children over several years, preferably into adolescence to examine the onset and course of psychological problems. Second, studies should investigate the relation between psychological and developmental problems, because they may reinforce each other. Third, the contribution of the postnatal and childhood environment ought to be explored, as some of the problems we detect may be worsened or improved by the family and kindergarten. The causal pathways, mediators, and moderators between early term/late preterm birth and emotional/behavioral problems should also be explored.

## Conclusions

Girls born late preterm and early term show an increased risk of emotional problems at 36 months of age. This finding suggests that gender should be taken into account when evaluating children born at these gestational ages.

## List of abbreviations

CBCL: Child Behavior Check List
CI: Confidence interval
IRT: Item response theory
ITSEA: Infant Toddler Social and Emotional Assessment
MBRN: Medical Birth Registry of Norway
MoBa: Norwegian Mother and Child Cohort Study
OR: Odds ratio
SCL-8: Hopkins Symptom Check List – 8 items
